# Self-Propelling Targeted Magneto-Nanobots for Deep Tumor Penetration and pH-Responsive Intracellular Drug Delivery

**DOI:** 10.1038/s41598-020-61586-y

**Published:** 2020-03-13

**Authors:** Saloni S. Andhari, Ravindra D. Wavhale, Kshama D. Dhobale, Bhausaheb V. Tawade, Govind P. Chate, Yuvraj N. Patil, Jayant J. Khandare, Shashwat S. Banerjee

**Affiliations:** 1 Maharashtra Academy of Engineering Education and Research’s Maharashtra Institute of Pharmacy, Pune, 411038 India; 2 Maharashtra Institute of Medical Education and Research, Talegaon Dabhade, Pune 410507 India; 3 School of Pharmacy, Dr. Vishwanath Karad MIT World Peace University, Pune, 411038 India

**Keywords:** Molecular machines and motors, Nanoparticles, Drug delivery

## Abstract

Self-propelling magnetic nanorobots capable of intrinsic-navigation in biological fluids with enhanced pharmacokinetics and deeper tissue penetration implicates promising strategy in targeted cancer therapy. Here, multi-component magnetic nanobot designed by chemically conjugating magnetic Fe_3_O_4_ nanoparticles (NPs), anti-epithelial cell adhesion molecule antibody (anti-EpCAM mAb) to multi-walled carbon nanotubes (CNT) loaded with an anticancer drug, doxorubicin hydrochloride (DOX) is reported. Autonomous propulsion of the nanobots and their external magnetic guidance is enabled by enriching Fe_3_O_4_ NPs with dual catalytic-magnetic functionality. The nanobots propel at high velocities even in complex biological fluids. In addition, the nanobots preferably release DOX in the intracellular lysosomal compartment of human colorectal carcinoma (HCT116) cells by the opening of Fe_3_O_4_ NP gate. Further, nanobot reduce *ex vivo* HCT116 tumor spheroids more efficiently than free DOX. The multicomponent nanobot’s design represents a more pronounced method in targeting tumors with self-assisted anticancer drug delivery for ‘far-reaching’ sites in treating cancers.

## Introduction

Designing miniaturized and versatile robots in the dimensional-range of a few micrometers or less offer potential for unprecedented biomedical applications, such as refinements in targeted drug delivery platforms^1–7^. Miniature robotic systems provide considerable benefits over conventional and micro/nanoparticle-based therapies^8,9^. Existing anticancer drug delivery systems demonstrate pharmacokinetic (PK) limitations as they are passive systems driven by the blood fluidics and lack intrinsic navigation for long circulation time, targeting, localized delivery, and tissue penetration^10,11^. Furthermore, despite surface functionalization with a specific ligand that allows nanocarriers to increase the active targeting ability; the nanocarriers are unable to guide themselves to a target. Hence, for targeted anticancer delivery of therapeutic payloads to disease sites, drug carriers are desired to possess some distinctive traits, including self-propelling force and velocity, navigational functions, precise cell targeting, drug cargo-towing and finally tissue penetration with the release of drug payload^12–16^.

Micro/nanomotors with efficient cargo towing and effective penetrating abilities make them excellent delivery vehicles that can meet the necessary features for targeted delivery of therapeutics^6^. Chemically propelled micro-/nanorobots have been widely explored for active drug delivery, and tremendous progresses has been made in the past few years^17^. However, designing nanobots for biological functionality is still a challenge as they have some inherent limitations, such as complex preparation technology, difficulty of surface modification, difficulty of motion in biological fluids and depending on the material, poor biocompatibility or biodegradability^6,18,19^. Furthermore, none of the reported micro/nanobot system has demonstrated practically useful speed high enough for biomedical applications due to high-speed blood flow in human arteries (dimensions from 4 to 25 mm) with a blood flow velocity from 100 to 400 mm/s^20^.

**Figure 1:**
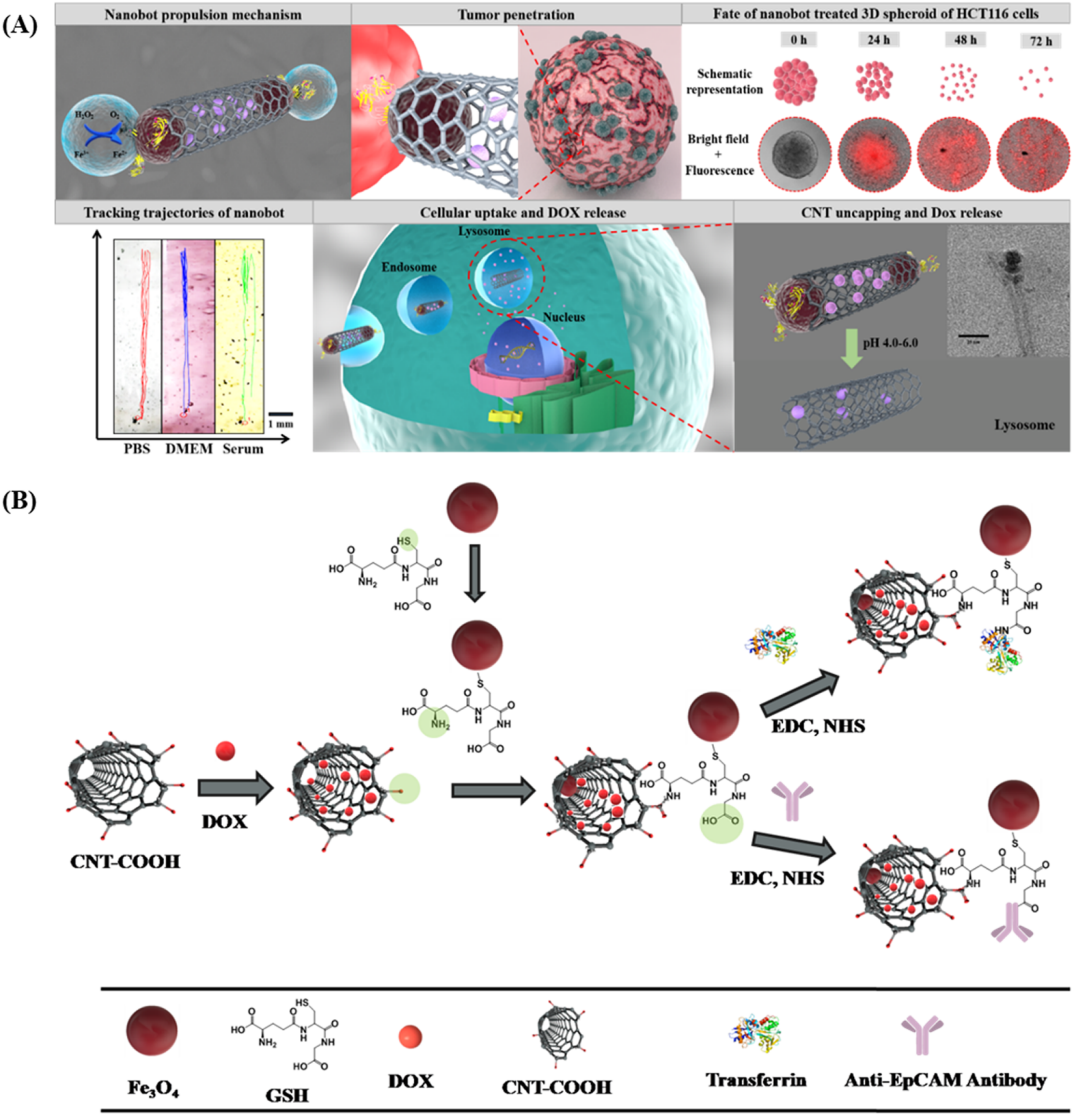
(**A**) Schematic representation of mechanism of oxygen bubble induced autonomous propulsion of nanobot and deep penetration in the tumor due to the generated thrust, fate of 3D spheroid treated with CNT-DOX-Fe_3_O_4_-Tf/CNT-DOX-Fe_3_O_4_-mAb nanobot, trajectories of nanobots in physiologically relevant media (trajectories obtained using Dino-Capture 2.0 v (https://www.dino-lite.com/), VirtualDub 1.10.4 v (http://www.virtualdub.org/) and MTrackJ plugin from ImageJ 1.8.0_112v (https://imagej.net/MTrackJ), followed by illustration of targeting DOX-loaded nanobot to transferrin/EpCAM receptor and entry in cancer cell, and finally, mechanism of triggered drug release under intracellular endo/lysosomal conditions. (**B**) Schematic illustration indicating the step-by-step synthesis of DOX loaded CNT-DOX-Fe_3_O_4_-Tf/ CNT-DOX-Fe_3_O_4_-mAb.

Herein, we report for the first time a smart H_2_O_2_ and pH-responsive nanobot system to transport anticancer drug deep inside the three dimensional (3D) tumors by exploiting Fe_3_O_4_ dependent decomposition of H_2_O_2_ existing in the tumor microenvironment (TME) into water and oxygen. Tumor cells are known to produce H_2_O_2_ at the rate of 0.5 nmol/10^4^ cells/h^21^. The nanobot was designed by chemically coordinating Fe_3_O_4_ NPs, conjugating anti-EpCAM mAb to carbon nanotubes (CNT) through reactive spacer glutathione (GSH) and loading of anticancer drug DOX. The unique advantages of anchoring Fe_3_O_4_ NPs are, as they impart autonomous propulsion ability and superparamagnetic property to the nanobot system. Further they also impart mechanism of “*on demand*” intracellular release of the encapsulated DOX. Thus, the Fe_3_O_4_ NP gates retard premature and non-specific release of DOX encapsulated in CNT thus minimizing therapy side effects. CNT platform was utilized as a carrier because it offers the benefit of chemical tunability, allowing integration of multiple component by conjugation chemistry including targeting moieties^22^. Importantly, functionalized CNTs have shown low toxicity and enhanced clearance, and even can be decomposed inside the human body^23^. CNTs with such advantages have been exploited to deliver various bioactive substances and contrasting agents. However, they have primarily been used as passive nanocarriers. Here, we have transformed passive CNTs into active autonomous nano-propelled-bots with controlled anticancer drug delivery platform, cellular specificity, targeting and deep 3D tumor penetration capability (Fig. 1A). Further, Fe_3_O_4_-catalyzed *in-situ* generation of oxygen from TME H_2_O_2_ may also help in relieving tumor hypoxia with potential augmentation of antitumor influence.

The present work, demonstrates a nanobot drug delivery platform that facilitates propulsion in biological fluids, cellular targeting, modulates the intracellular release and enhanced penetration to TME for improved anti-cancer therapy.

## Results and discussion

### Antibody/Tf-targeted nanobot conjugation and characterization

Tf and anti-EpCAM mAb conjugated nanobots were designed by multi-step chemical conjugation process (Fig. 1B). CNTs were first subjected to oxidation treatment to create abundant carboxylic groups mostly at the tips and defect sites of CNT surfaces. DOX was successfully encapsulated in the hollow CNTs (with inner diameter of ~11 nm) as the inner surface is hydrophilic, and aqueous solutions containing DOX can be loaded inside through the open ends. Here, we hypothesize that loading of DOX in CNTs will protect it from the early exposure to physiological milieu. Further, Fe_3_O_4_ NP was conjugated to DOX loaded CNT through the GSH linker by the EDC coupling method. Thereafter, anti-EpCAM mAb was conjugated to the surfaces of CNT by EDC coupling reaction using the carboxyl groups on the CNT resulting in CNT-DOX-Fe_3_O_4_-mAb nanobots. Similarly, Tf was conjugated to the reactive surface of CNT resulting in CNT-DOX-Fe_3_O_4_-Tf nanobots. Tf protein has been used as a model targeting moiety to the cancer cells with overexpressed Tf receptors (TfR^+^).

**Figure 2:**
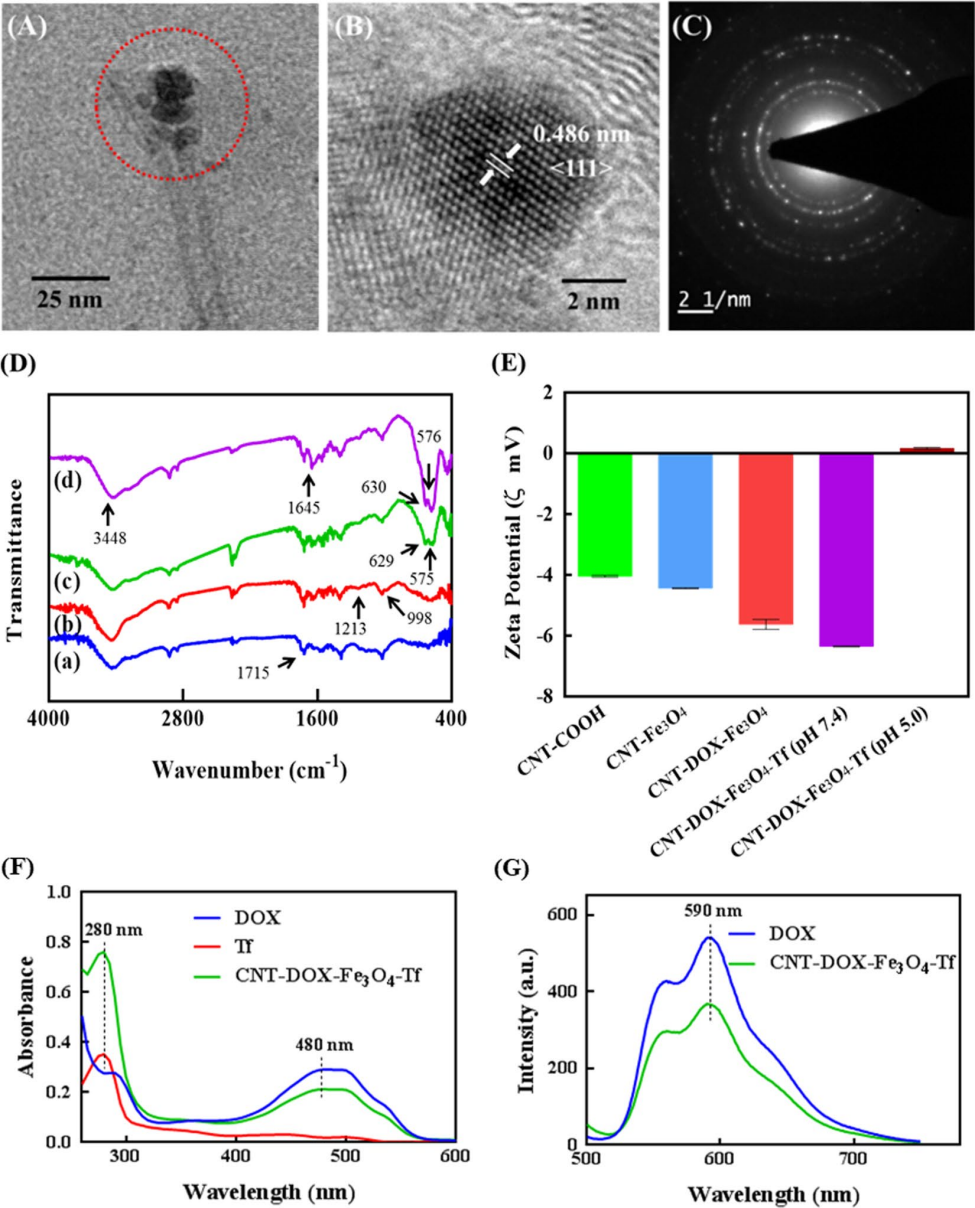
Characterization of CNT-DOX-Fe_3_O_4_-Tf and CNT modifications to obtain the multicomponent CNT-DOX-Fe_3_O_4_-Tf (nanobot). (**A**) TEM microscopy images of CNT-DOX-Fe_3_O_4_-Tf**, (B**) evidencing Fe_3_O_4_ structure, and (**C**) crystalline features of the NPs. (**D**) FTIR spectra of of (a) CNT-COOH, (b) CNT-DOX, (c) CNT-DOX-Fe_3_O_4_ and (d) CNT-DOX-Fe_3_O_4_-Tf. (**E**) surface charge evolution upon loading of CNT with DOX and further conjuagtion of Fe_3_O_4_ and Tf, (**F**) UV-visible spectra of DOX (λ_max_ = 480 nm), Tf (λ_max_ = 280 nm) and CNT-DOX-Fe_3_O_4_-Tf (Tf peak at 280 nm and DOX peak at 480 nm). (**G**) Normalized fluorescence spectra of DOX and CNT-DOX-Fe_3_O_4_-Tf (λ_ex_ = 480 nm, λ_em_ = 590 nm).

Transmission electron microscope (TEM) images of CNT-DOX-Fe_3_O_4_-Tf nanobot revealed the presence of spherical Fe_3_O_4_ NPs of average diameter ~16 nm at the tip ends of CNTs (Fig. 2A and Supplementary Fig. S1). Crystallographic structure of the Fe_3_O_4_ NPs analyzed by high resolution TEM (HRTEM) showed magnetite crystalline nature (Fig. 2B). Furthermore, the identified lattice fringes co-related well to the structure of magnetite planes with a plane-to-plane separation of 0.486 nm. The Selected Area Electron Diffraction (SAED) pattern revealed spotty diffraction rings and well resolved spots thus confirming crystalline Fe_3_O_4_ structure for the conjugated NPs (Fig. 2C).

The CNT-DOX-Fe_3_O_4_-Tf nanobot was also characterized by FTIR to verify the successful covalent conjugation between CNT, Fe_3_O_4_ and Tf. Figure 2D shows the FTIR spectra of oxidized CNT, CNT-DOX, CNT-DOX-Fe_3_O_4_ and CNT-DOX-Fe_3_O_4_-Tf, respectively. The IR spectrum of CNT showed characteristic peak at 1715 cm^−1^ due to the presence of carbonyl groups. DOX loaded CNT showed characteristic peaks of DOX at 998 cm^−1^ and 1213 cm^−1^ indicating presence of DOX in CNT. The IR spectrum of CNT-DOX-Fe_3_O_4_ showed prominent peaks at 575 cm^−1^, 629 cm^−1^ due to Fe-O stretching thus confirming the conjugation of GSH-Fe_3_O_4_ to the CNT^21,24,25^. Furthermore, the spectrum of CNT-DOX-Fe_3_O_4_ conjugated with Tf showed new peaks at 3448 cm^−1^ for free amine, and sharp peak at 1645 cm^−1^ for amide linkage, providing clear evidence for conjugation of Tf with CNT-DOX-Fe_3_O_4._ We also evaluated the conjugation reaction with respect to the change in zeta potential of the individual step during the synthesis of CNT-DOX-Fe_3_O_4_-Tf (Fig. 2E). The zeta potentials of CNT-COOH, Fe_3_O_4_, CNT-DOX-Fe_3_O_4_ and CNT-DOX-Fe_3_O_4_-Tf were determined to be −4.07, −18.6, −8.9, and −22.2 mV, respectively. The step-wise altered zeta potentials indicated successful conjugation of the multiple components with CNT. Tf conjugation quantified by a modified Bradford procedure was found to be ∼326 mg per g of CNT-DOX-Fe_3_O_4_.

The drug loading and encapsulation efficiency of DOX was determined to be 63.8 µg/mg in CNT-DOX-Fe_3_O_4_ nanobots using UV-visible spectrophotometry. DOX loading and Tf conjugation in CNT-DOX-Fe_3_O_4_-Tf was analyzed and confirmed by UV-visible and fluorescence spectroscopy methods. The UV-visible spectrum of CNT-DOX-Fe_3_O_4_-Tf was compared with the spectra of free DOX and Tf (Fig. 2F). The spectra revealed the presence of characteristic peaks of DOX (λ_max_ = 480 nm) and Tf (λ_max_ = 280 nm) in the CNT-DOX-Fe_3_O_4_-Tf nanobots. Furthermore, the fluorescence spectrum of the CNT-DOX-Fe_3_O_4_-Tf was compared to that of the free DOX under identical optical conditions (480 nm excitation). As depicted in Fig. 2G, typical DOX in PBS displayed λ_em_ at ~590 nm. The spectrum of CNT-DOX- Fe_3_O_4_-Tf also displayed the typical absorption band from DOX indicating loading of DOX. In addition, the presence of DOX in CNT was also confirmed using 2.5D fluorescence microscopy imaging of the CNT-DOX-Fe_3_O_4_ nanobots. The image revealed presence of DOX (red) within the nanopores of the CNT carrier particle (gray) (Supplementary Fig. S2).

### Motion and position-kinetic analysis of nanobots

**Figure 3:**
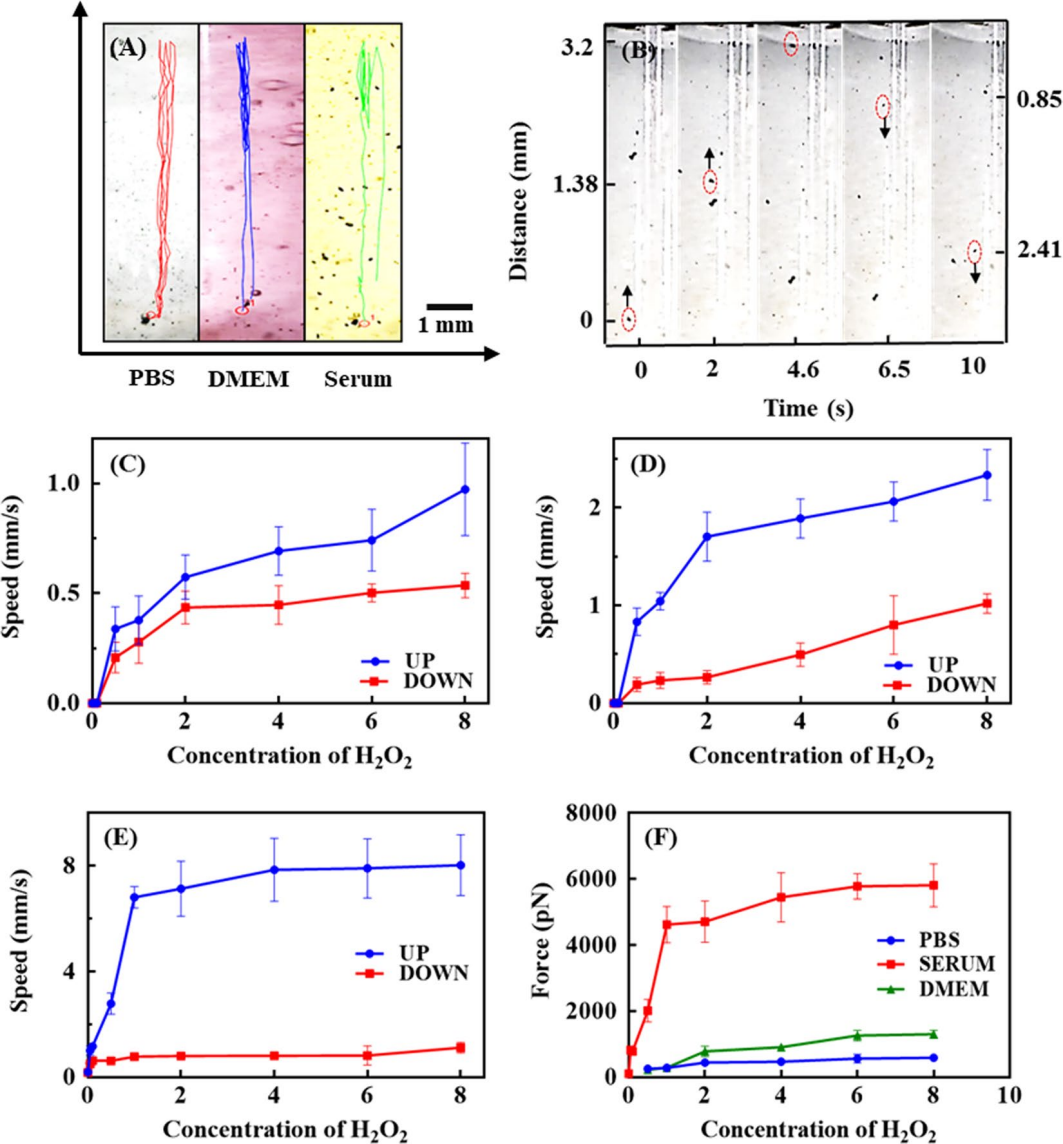
(**A**) Analysis of the motion behavior of CNT-DOX-Fe_3_O_4_-Tf nanobot. The videos were recorded with Dino-Lite digital microscope at 50× magnification, using the Dino-Capture 2.0 v (https://www.dino-lite.com/), best clip was chosen using VirtualDub 1.10.4 v (http://www.virtualdub.org/) and finally tracking and speed calculations were performed using MTrackJ plugin from ImageJ 1.8.0_112v (https://imagej.net/MTrackJ). (a) Representative tracking trajectories of CNT-DOX-Fe_3_O_4_-Tf nanobots with different biologically relevant media. (**B**) Time-lapse images of the nanobot driven by oxygen bubble propulsion after time intervals of (a) 0, (b) 2.0, 4.6, 6.5 and 10 s. Speed of nanobot in the presence of different concentration of H_2_O_2_ (0.5–8 w/v %) in (**C**) PBS, (**D**) DMEM and (**E**) serum, (**F**) Analysis of force of nanobot in PBS, DMEM and serum in presence of different concentration of H_2_O_2_ (0.5–8 w/v %).

The self-propelling abilities of the CNT-DOX-Fe_3_O_4_-Tf nanobot in different fluids simulating physiological environments such as in phosphate buffer saline (PBS; pH 7.4), Dulbecco’s modified eagle medium (DMEM) cell media and serum were characterized to verify the compatibility in relevant biological fluids. Some organic and/or biological molecules are capable of quenching or inhibiting the H_2_O_2_ decomposition reactions catalyzed by Fe_3_O_4_ NPs and thus can significantly hamper the motion of the nanobot. NP tracking analysis was used to track in real-time the movement of the nanobots under a range of H_2_O_2_ concentrations (Fig. 3A). The nanobots propelled upward instantaneously and gradually reverted in the downward direction. For the mechanism of motion, O_2_ bubbles generated by Fe_3_O_4_ NPs catalyzed decomposition of H_2_O_2_ are responsible for propulsion in this system. The catalytic ability of Fe_3_O_4_ evaluated in PBS comprising a range of H_2_O_2_ (0.006 w/v% to 0.05 w/v%) concentrations revealed increased rate of reaction with increase in H_2_O_2_ concentration (Supplementary Fig. S3). Supplementary Fig. S4 shows propelling CNT-DOX-Fe_3_O_4_-Tf nanobots in PBS buffer at pH 7.4 with 0.5% H_2_O_2_ composition (Supplementary Fig. S4A) and its response when held next to a permanent magnet (Supplementary Fig. S4B). CNT-DOX-Fe_3_O_4_-Tf nanobots moving in vertical trajectory was acquired through the solution and got accumulated at the side of the tube where the magnetic field gradient was the strongest. Hence, the direction of the nanobots can be remotely controlled by a magnetic field and thus enabling it a cooperative propulsion mode under magnetic field in the presence of the chemical fuel.

Figure 3B shows images of the nanobot at different positions during its motion for a complete cycle. As evident from the images, the nanobot stayed away from the wall and moved through nearly the center of the liquid column during its flight. The average propulsion speed of the CNT-DOX-Fe_3_O_4_-Tf nanobot during its upward movement velocity in PBS, DMEM, and the blood serum was 0.338, 0.831 and 1.011 mm s^−1^ respectively, in 0.5% H_2_O_2_. On the other hand, the downward velocity of nanobots was measured to be 0.208, 0.221 and 0.502 mm s^−1^, respectively. The velocity and speed of nanobots was virtually stable without obvious deceleration for more than 5 cycles.

Interestingly, the upward and downward velocity of the nanobot in PBS, DMEM, and serum increased significantly to 0.972, 2.333, 8.026 mm s^−1^ (equal to a relative speed of nearly 119 body length per second) and 0.535, 1.120, 1.120 mm s^−1^ when the concentration of H_2_O_2_ increased to 8% H_2_O_2_ (Fig. 3C–E). This corresponds to a large driving force of 592, 1304 and 5435 pN in the upward direction, based on the drag force F = 6π*μ*rv, where v is the speed, r is the radius of the nanobot and *μ* is the viscosity of the medium (Fig. 3F). The increase in speed with increasing H_2_O_2_ concentration is due to influence of surrounding H_2_O_2_ concentration on the reduction rate of the Fe^3+^ to Fe^2+^. Hence, with the presence of higher localized concentration of H_2_O_2_ lead to an increased production of O_2_ bubbles thus resulting in generation of strong thrust and buoyancy thereafter for the upward as well downward motion of the nanobots (Fig. 3F). Further, the speed of the nanobot in serum was ~8.3 and ~3.4 times the speed seen in PBS and DMEM. The distance travelled by the nanobot in serum changed with change in H_2_O_2_ concentration. At low H_2_O_2_ concentration (0.5%) the average distance travelled was low (19.069 mm), while it was high (63.543 mm) at higher concentration (8%). The three-fold enhancement of distance travelled by nanobots was influenced due to innate H_2_O_2_ present in blood. H_2_O_2_ has diverse roles in normal physiological context. It serves as a blood borne signaling molecule, while at the same time it is produced intra-mitochondrially in most live cells. While these sources produce small amount of H_2_O_2_, the circulatory system conceivably accumulates this product. Additionally, immune cells, endothelial, and unbound xanthine oxidase generate H_2_O_2_ which also increase the cumulative H_2_O_2_ serum levels^26,27^. Serum H_2_O_2_ content varies between 0–5 µm depending on physiological conditions^28^. Significantly, tumor cells influence H_2_O_2_ content locally and presumably systemically^29–31^. Tumors are known to demonstrate the capability of exploiting H_2_O_2_ in cell proliferation^32^. However, a restrained capacity to metabolize H_2_O_2_ drives tumor masses to drain nascent H_2_O_2_ in the surrounding tissue space which may ultimately reach systemic circulation and may increase systemic levels by up to 10 µm and higher^33^. Further, the catalase enzyme present in serum may also be imparting catalytic property by getting adsorbed on the surface of nanobots and thus greatly enhancing generating of oxygen bubbles. In addition, it is conceivable that as a result of localized protein oxidation in the presence of H_2_O_2,_ the protein aggregation leads to the adsorption of serum proteins such as albumin and immunoglobulins on the surface of the NPs^34,35^. Aggregated proteins have a cascading effect which may further influence binding of other serum proteins including enzymes such as serum catalase onto the surface of the protein-masked NPs^36^. This synergistic effect may also be responsible for the rapid propulsion of nanobots in blood serum even at low H_2_O_2_ concentration as compared to PBS and DMEM^4,36–40^. The results indicate an appropriate pairing of the propulsion mechanism pre-assumed for its physiological fate and subsequently for the clinical context. It may be possible to exploit the natural H_2_O_2_ decomposition system in combination with limited exogenous H_2_O_2_ and attain high propulsion resulting in significant driving force to nanobots for rapid transport of drug cargo followed by deep tumor penetrating capability.

### Drug release profiles of the nanobots

To investigate the pH dependent control release of DOX, we performed drug release study at two different pH conditions, one representing the physiological pH i.e. 7.4 and the other cell lysosomal pH (~pH 5) in presence and absence of proteases enzyme-cathepsin B. As shown in Supplementary Fig. S5, CNT-DOX-Fe_3_O_4_-Tf nanobot demonstrated low release of DOX (~26%) even after 48 h at pH 7.4, signifying efficient trapping of DOX in the CNT cavities by with Fe_3_O_4_ NPs exterior cap. The observed small DOX release is probably of the loosely surface-bound DOX. Conversely at pH 5 and in presence of cathepsin B, a controlled DOX release pattern was observed. Around ~76% DOX got released till 4 h which then increased to ~94% at 48 h. This remarkable multi-order kinetics pattern of DOX release from the designed nanobot is due to the degradation of amide linkage resulting in time-dependent uncapping of CNT^41^. TEM images of nanobots after release study confirmed the uncapping of CNTs as no Fe_3_O_4_ NPs were seen near the tip of the CNTs (Supplementary Fig. S6). However, in absence of cathepsin B ~75% DOX got released till 48 h in pH 5.0. The release of DOX is most likely due to the degradation of amide linkage in acidic pH^42^. Similarly, at pH 6.5 and in presence of cathepsin B, ~85% DOX got released till 48 h. However, in absence of cathepsin B only ~56% DOX got released. Further, to confirm the capping efficiency, release of DOX from CNT-DOX-Fe_3_O_4_-Tf nanobot without the Fe_3_O_4_ NP cap was also examined. Nanobot without cap demonstrated a predictable immediate burst-release of ~61% and ~18% of DOX within 30 min in pH 5.0 and 7.4, respectively. As mentioned earlier, the open-ended CNT allow cross-flow in the CNT cavity and consequently allow rapid release of the entrapped DOX. This pH-sensitive release behavior is of particular interest as it can reduce untimely drug release during systemic circulation and can specifically enhance intracellular (lysosomal) DOX release. This will be beneficial in cancer treatment as it will help in significantly lowering the dosage, few side effects and limited drug toxicity.

### Time dependent cell entry kinetics studies

**Figure 4:**
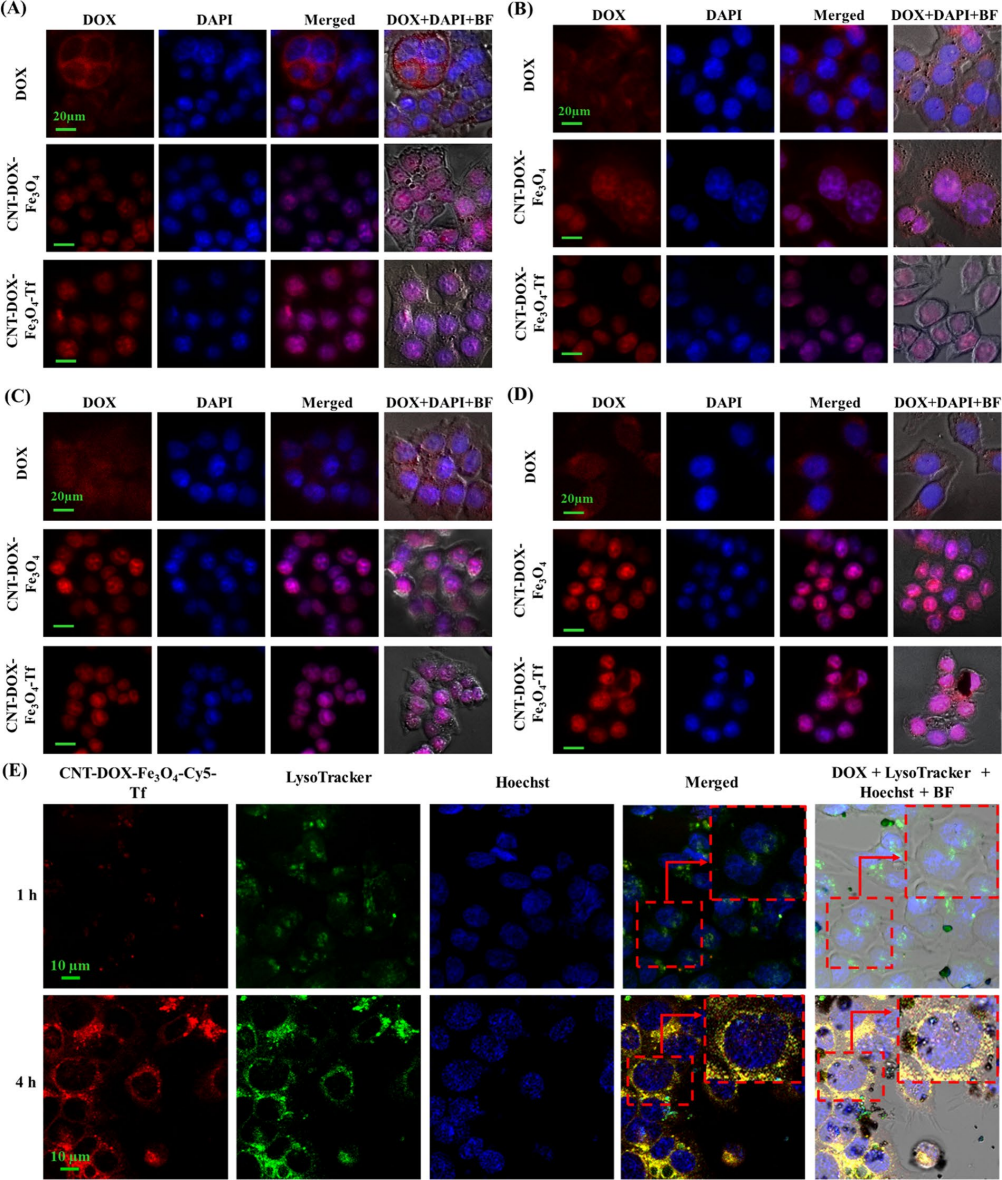
Fluorescent images of HCT116 cells treated with free DOX, CNT-DOX-Fe_3_O_4_ and CNT-DOX-Fe_3_O_4_-Tf. (**A**) At 4 h exposure and at pH 7.4, DOX released from CNT-DOX-Fe_3_O_4_ and CNT-DOX-Fe_3_O_4_-Tf was observed to be localized in the nuclear region (**A,B**). The intracellular release of DOX can be attributed to the opening of pH-sensitive nanogates due to amide bond cleavage in the acidic lysosomal compartments. Cells incubated with free DOX showed efflux of DOX from the nucleus back into the cytoplasm, which is in contrast to the findings for CNT-DOX-Fe_3_O_4_ and CNT-DOX-Fe_3_O_4_-Tf. (**B**) At 4 h exposure and at pH 6.5, the fluorescence intensity of DOX from CNT-DOX-Fe_3_O_4_-Tf nanobot was higher due to faster cellular internalization of CNT-DOX-Fe_3_O_4_-Tf. (**C**) At 24 h and at pH 7.4, most of the DOX was released from CNT-DOX-Fe_3_O_4_-Tf suggesting the efficient release of DOX from interior cavity of CNT after opening of Fe_3_O_4_ nanogate in lysosomal conditions. (**D**) At 24 h and at pH 6.5, the fluorescence intensity of DOX in the cells was more pronounced suggesting enhanced cellular internalization of CNT-DOX-Fe_3_O_4_-Tf nanobot (Scale bars indicate 20 μm). (**E**) Kinetic study of Fe_3_O_4_ NP uncapping and DOX release from CNT-DOX-Fe_3_O_4_-Cy5-Tf nanobots in cells using confocal microscopy. Time-dependant release of DOX (red) into the acidic lysosomal compartment (green, LysoTracker) over 4 h, indicating -cleavage of CNT- Fe_3_O_4_ amide-bond, subsequent uncapping and DOX release. The merged image of the cells at 4 h shows a prominent yellow-orange signal indicating co-localization of DOX and lysosomes around the nucleus (blue), scale bars indicate 10 µm.

**Figure 5:**
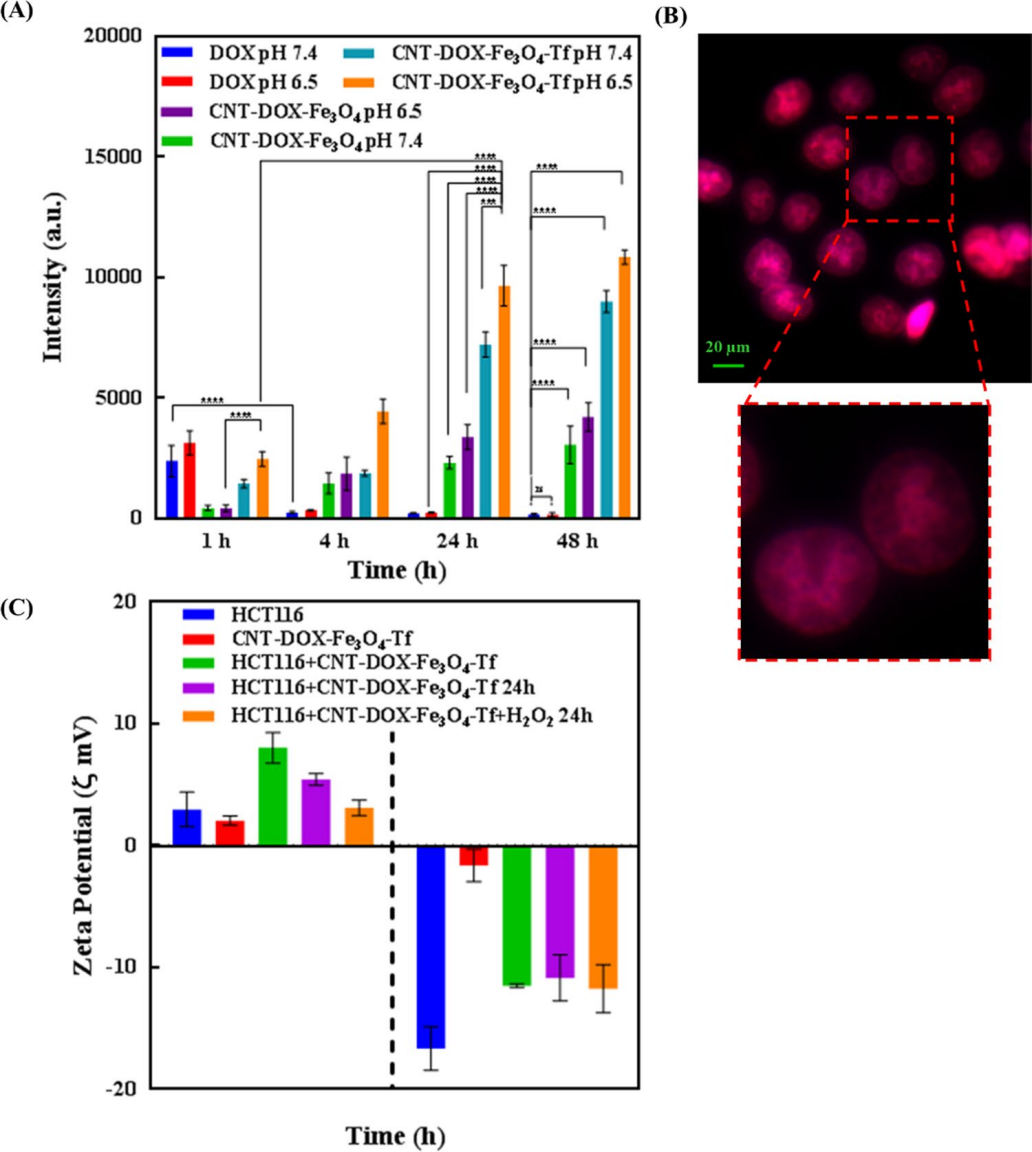
(**A**) Fluorescence intensity of intracellular DOX accumulation upon treatment with nanobots at varying pH. (**B**) DOX binding of nucleoli. The nucleolar enrichment of DOX post NP administration is suggestive of high-affinity binding of DOX to nucleoli. (**C**) Surface charge evolution upon exposing to CNT-DOX-Fe_3_O_4_-Tf in presence and absence of H_2_O_2_.

The cellular uptake and intracellular pH-dependent endo/lysosomal release of DOX from nanobots was studied over time by fluorescent cell imaging (Fig. 4). HCT116 colon cancer cells were cultured, and subsequently incubated with DOX, CNT-DOX-Fe_3_O_4_ and CNT-DOX-Fe_3_O_4_-Tf at 37 °C before examination under fluorescence microscope at definite time intervals. The inherent fluorescence emissions of DOX were red, which were utilized as indicators for their corresponding distribution inside the cells (Fig. 5A). Figure 4 and Supplementary Fig. S7 depict the entry of free DOX influx, CNT-DOX-Fe_3_O_4_ and CNT-DOX-Fe_3_O_4_-Tf into HCT116 cells, implied by rapid cytosolic DOX labeling followed by DOX importation into the nucleus. At the 1 h, CNT-DOX-Fe_3_O_4_ and CNT-DOX-Fe_3_O_4_-Tf internalized into the cells by mechanisms including endocytosis and energy-independent, direct penetration and were localized mainly in the cytoplasm and subcellular vesicles. The (DAPI-stained) nucleus displayed a low DOX presence as compared to the cytosolic compartment (Supplementary Fig. S7A). Interestingly, the emission of DOX overlapped exactly with that of CNT-DOX-Fe_3_O_4_-Tf. In contrast, cells treated with free DOX showed red fluorescence accumulation mainly in the cell nuclei. Exposure of the cancer cells to free DOX resulted in rapid influx owing to passive diffusion as well as carrier-mediated uptake of DOX^43^. The fluorescence intensity of free DOX in the cell was ~1.7 times higher than that of CNT-DOX-Fe_3_O_4_-Tf. On the other hand, the intensity of DOX released from CNT-DOX-Fe_3_O_4_ was 3.3 times less than CNT-DOX-Fe_3_O_4_-Tf. The influx of DOX into the nucleus is believed to be facilitated by binding to proteasomes^44,45^. On the other hand, energy-dependent drug efflux mechanisms such as ATP-binding cassette subfamily C member 1 (ABCC) are implicated in active efflux of DOX out of the cell^46^. The efflux machinery in turn contributes to the drug resistance of cancer cells. Furthermore, to understand how the TME affect the nanobot internalization process and intracellular delivery of DOX, the cellular entry kinetics was also studied at an acidic pH of 6.5. The pH of the media showed a clear influence on nanobot cell internalization and intracellular DOX release. While the CNT-DOX-Fe_3_O_4_ nanobot showed comparable DOX presence at 1 h in both pH environments, the CNT-DOX-Fe_3_O_4_-Tf nanobot demonstrated ~1.7 fold increase in cellular DOX content in the acidic pH of 6.5, compared to the normal physiological pH 7.4 (Supplementary Fig. S7B, and Fig. 5A). The study clearly reveals higher cell entry of CNT-DOX-Fe_3_O_4_-Tf nanobot at pH 6.5.

After incubation for 4 h, DOX released from CNT-DOX-Fe_3_O_4_ and CNT-DOX-Fe_3_O_4_-Tf was observed to be localized in the nuclear region (Fig. 4A,B). The intracellular release of DOX can be attributed to the opening of pH-sensitive nanogates due to amide bond cleavage in the acidic lysosomal compartments (Fig. 1). Additionally, the release of DOX was studied using confocal laser scanning microscopy (CLSM). At 4 h the LysoTracker labeled acidic organelles appeared yellow-orange, owing to merging of the green (LysoTracker) and red (DOX) fluorescence, due to the release of DOX from CNT-DOX-Fe_3_O_4_-Tf (Fig. 4E). Subsequently, to further confirm the uncapping of CNT-DOX-Fe_3_O_4_-Tf nanobots, Fe_3_O_4_ NPs in CNT-DOX-Fe_3_O_4_-Cy5-Tf were labeled with a fluorescent dye, Cyanine 5 (Cy5). As depicted in Supplementary Fig. S8, a strong localization of Cy5 (purple signal, Fe_3_O_4_ NPs) with DOX (red) at 1 h was suggestive of site-restriction of DOX within CNT-DOX-Fe_3_O_4_-Cy5-Tf nanobots. However, in 4 h post-treatment images the whole LysoTracker labelled acidic organelles appeared orange indicating separation of Fe_3_O_4_ and DOX signals, consistent with detachment of Fe_3_O_4_ caps from CNT and subsequent release of DOX from CNT. This finding is consistent with the DOX release patterns from CNT-DOX-Fe_3_O_4_ and CNT-DOX-Fe_3_O_4_-Tf at pH 5.0 (Supplementary Fig. S5). The fluorescence intensity of DOX for CNT-DOX-Fe_3_O_4_-Tf was ~8 times higher than that of free DOX (Fig. 5A). Cells incubated with free DOX showed efflux of DOX from the nucleus back into the cytoplasm, which is in contrast to the findings for CNT-DOX-Fe_3_O_4_ and CNT-DOX-Fe_3_O_4_-Tf. Efflux of DOX prior to its activity in arresting topoisomerase is likely the reason for reduced efficacy of DOX. A rapid back-efflux phenomenon indicated an adaptive mechanism for drug resistance. It is conceivable that the efflux transport of free DOX occurs at a significantly higher velocity than that afforded by the DOX-proteasome nuclear import mechanism. On the other hand, the fluorescence intensity of DOX at pH 6.5 from CNT-DOX-Fe_3_O_4_-Tf nanobot was ~2.4 times higher than that observed in pH 7.4 (Figs. 4B and 5A). The presence of higher DOX could be attributed to faster cellular internalization of CNT-DOX-Fe_3_O_4_-Tf in pH 6.5 as compared to pH 7.4.

Tf is a vital protein for cellular uptake of systemic iron, consequently, the receptor mediated endocytosis which drives the import of exogenous CNT-DOX-Fe_3_O_4_-Tf nanobot ensures the capture, internalization, processing and release of DOX intracellularly. While diffusion of DOX and transporter mediated DOX import appears faster in the free DOX state, CNT-DOX-Fe_3_O_4_-Tf seemingly maintains molecular efficiency in DOX import^47^. Put differently, the deficiency of the Tf-conjugated nanobot in rapid initial diffusion velocity, as seen in free DOX, is compensated by the sustained import of Tf-nanobot-borne DOX. It is possible that the endosomal processing of nanobot-encapsulated DOX results in efficient presentation of liberated DOX to cellular proteasomes which in turn deliver it to the nucleus. In contrast, the CNT-DOX-Fe_3_O_4_-borne DOX is introduced within the cell in a diffusion and energy-independent membrane flipping manner. Presumably this exposes the DOX to cellular environment and therefore the efflux machinery resulting in poorer DOX nuclear import as compared to the CNT-DOX-Fe_3_O_4_-Tf nanobots.

At 24 h, DOX was almost exclusively present in the nucleus of the cells treated with the CNT-DOX-Fe_3_O_4_ and CNT-DOX-Fe_3_O_4_-Tf nanobots (Fig. 4C,D). Post-endosomal and lyzosomal processing and Fe_3_O_4_ amide-bond cleavage, the released DOX undergoes the same nuclear entry pathway as free DOX, i.e. *via* proteasomes. However, the 24 h retention of DOX within the nuclear compartment is a significant improvement over free DOX. The nuclear efflux is apparently low or virtually non-existent in case of CNT-DOX-Fe_3_O_4_ and CNT-DOX-Fe_3_O_4_-Tf, which is further evidenced by a virtual absence of DOX from the cytoplasm. Strongly contrasted with this nanobot-borne DOX behavior is the gradual disappearance of DOX from the cellular compartments in cells treated with free DOX. The fluorescence intensity of DOX for CNT-DOX-Fe_3_O_4_-Tf was ~35 times higher than free DOX (Figs. 4C and 5A). It is conceivable that the DOX, free from the influence of nanobot-mediated outcomes, is rapidly effluxed from the cell. ATP-dependent ABCC1 drug transporter is postulated to work even against the DOX concentration gradient across the cell membrane and achieve high DOX clearance. Interestingly, at pH 6.5, the fluorescence intensity of DOX in the cells exposed to CNT-DOX-Fe_3_O_4_-Tf nanobot was more pronounced than in pH 7.4. The intensity was ~1.3 times more in pH 6.5 suggesting enhanced cellular internalization of CNT-DOX-Fe_3_O_4_-Tf nanobot at pH 6.5 (Figs. 4B and 5A). The presence of higher DOX could be attributed to faster cellular internalization of CNT-DOX-Fe_3_O_4_-Tf in pH 6.5 as compared to pH 7.4.

At the 48 h, most of the DOX resided in the nuclei of the cells treated with CNT-DOX-Fe_3_O_4_ and CNT-DOX-Fe_3_O_4_-Tf (Supplementary Fig. S7C,D), similar to the outcome seen at 24 h. While nuclear retention was apparent for both treatments, DOX intensity appeared greater for CNT-DOX-Fe_3_O_4_-Tf indicating efficient and steady release of DOX from the target-specific CNT-DOX-Fe_3_O_4_-Tf nanobot. The amount of DOX effluxed from the cell was as high as 93% as determined from the kinetic study for free DOX. While the efflux kinetics for the free DOX was similar in the both the pH conditions, CNT-DOX-Fe_3_O_4_-Tf nanobot demonstrated pH sensitivity even at 48 h. As shown in Supplementary Fig. S7C,D, DOX released from CNT-DOX-Fe_3_O_4_-Tf nanobot co-localized with DAPI concentrated in the nuclear region highlighting the nucleosome bodies, which contain the chromatin matter. The effect is more pronounced at pH 6.5 and the DOX accentuation in the nucleus suggests preferential binding of DOX to DNA and nucleosome-bound topoisomerases (Fig. 5B). The consequence of targeted delivery of DOX using the CNT-DOX-F_3_O_4_-Tf vehicle was the inversion of the net efflux kinetics seen in free drug to the net accumulation kinetics of DOX when administered *via* targeted nanobots (Supplementary Fig. S9).

As mentioned earlier, while the efflux velocity of the free DOX may overcome its nuclear entry, the proteasome-facilitated DOX nuclear import may be instrumental in enhanced DOX entry into the nucleus when cells are treated with DOX-nanobots. Moreover, the CNT-DOX-Fe_3_O_4_ borne DOX may have secondary roles in enhanced nuclear delivery and nuclear retention which allow DOX to show nuclear presence past the clearance period for free DOX (Supplementary Fig. S7C,D). The proposed nanobot thus present a mechanism for evading drug efflux in cancerous cells and ensuring drug accumulation to achieve its cytotoxic goal.

To highlight the role of TME acidic milieu and H_2_O_2_ in the uptake of nanobots, zeta potential of the cells exposed to CNT-DOX-Fe_3_O_4_-Tf nanobot was evaluated at two different pH conditions, physiological pH 7.4 and pH 6.5 which exists in TME in presence of H_2_O_2_ as shown in Fig. 5C. The HCT116 cells demonstrated a negative surface charge in pH 7.4. However, at lower pH values (pH 6.5), the cells underwent surface charge modifications and exhibited a predominantly positive charge due to protonation of free fatty acid head groups in the outer lipid^48^. Furthermore, cells exposed to CNT-DOX-Fe_3_O_4_-Tf nanobot resulted in a significant alteration of the cell’s surface charge, regardless of the pH conditions. The zeta potential of the cells exposed to CNT-DOX-Fe_3_O_4_-Tf nanobot in pH 6.5 was roughly 3-times higher as compared to the cells alone. The increase in zeta potential of the cells can likely be attributed to surface-attachment of the nanobot which are also positively charged in acidic pH of 6.5. The increase in surface charge of the cells is shown to be reduced over time (24 h) and furthermore by co-incubation with H_2_O_2_ in acidic media. This may be interpreted as a gradual reduction in surface charge due to internalization of the nanobots by receptor-mediated endocytosis. In the presence of H_2_O_2_ at 24 h, the cells exposed to CNT-DOX-Fe_3_O_4_-Tf nanobot demonstrated a restoration to the initial zeta potential in acidic condition. It may be due to near-complete internalization of the attached CNT-DOX-Fe_3_O_4_-Tf nanobot in the cell. The acidic condition may have played a role in uptake of nanobots which is further accentuated in the presence of H_2_O_2_ as shown in Fig. 5C. Interestingly, the cells exposed to the CNT-DOX-Fe_3_O_4_-Tf nanobot at physiological pH did not show any major change over time or by the presence of H_2_O_2_ suggesting a slow internalization under physiological conditions. In accordance with the cell kinetics images (Fig. 4A–D), HCT116 cells do show greater DOX accumulation in acidic conditions.

### Nanobot’s efficacy as a drug delivery vehicle

**Figure 6:**
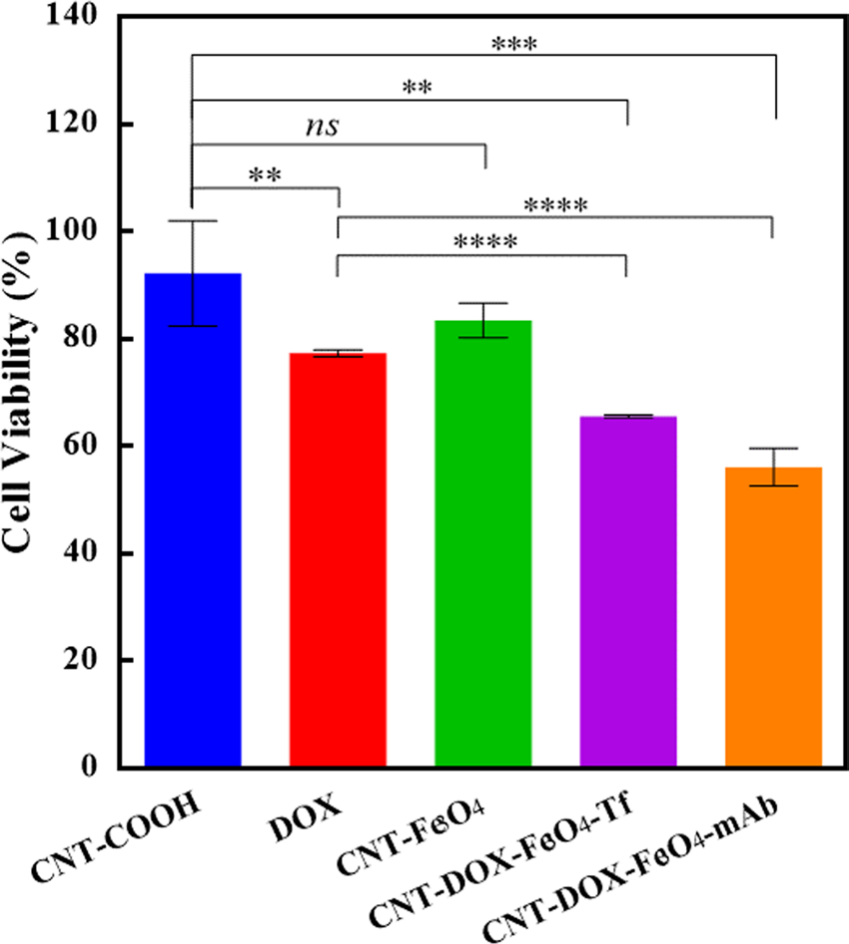
Cytotoxicity analysis of free DOX, CNT-DOX-Fe_3_O_4_, CNT-DOX-Fe_3_O_4_-Tf and CNT-DOX-Fe_3_O_4_-mAb nanobots incubated for 48 h with HCT116 cells. Cell viability study of treatments with free DOX and nanobots reveals a statistical improvement of CNT-DOX-Fe_3_O_4_-Tf and CNT-DOX-Fe_3_O_4_-mAb nanobots over free DOX treatment of HCT116 cells. The CNT-Fe_3_O_4_ nanobot does not show greater cytotoxic effect as compared to the control-CNT treatment. The free DOX shows limited toxicity to the model cancer cells at the end of the treatment. In contrast, the CNT-DOX-Fe_3_O_4_-Tf and CNT-DOX-Fe_3_O_4_-mAb nanobot loaded with an equivalent dose of DOX shows statistically significant improvement in the toxicity induced, suggesting greater efficacy of the DOX delivery by nanobot.

Concurring with the microscopy data presented, cell viability assays were performed to compare the cytotoxic effects of DOX, CNT-Fe_3_O_4_, CNT-DOX-Fe_3_O_4_-Tf and CNT-DOX-Fe_3_O_4_-mAb nanobot, show anticancer effect of the targeted nanobots. The control treatment with CNT (CNT-COOH) showed no cytotoxicity in the treated HCT116 cells. CNT-Fe_3_O_4_ nanobot showed a mild influence on decreasing viability of treated cells, however there was no statistical difference in the effects of control CNT and CNT-Fe_3_O_4_ as shown in the Fig. 6. DOX on the other hand showed anticancer effect in HCT116 cells, based on the reduced viability of treated cells. The reduced cytotoxicity of the topoisomerase inhibitor *viz a viz* drug is attributed to the activity of the efflux pump which drive DOX out of the cell and decrease its intercalation with DNA^33^. As also seen in the cellular kinetics study (Fig. 4), DOX rapidly localizes to the nuclear region, however the energy-dependent efflux pumps are credited with effective removal of DOX from the nuclear compartment and eventually the cytoplasm as well. In contrast the targeted nanobots demonstrated superior nuclear DOX retention and maintained nuclear localization of the DOX for up to 48 h. The greater cytotoxicity of the targeted CNT-DOX-Fe_3_O_4_-Tf/mAb and CNT-DOX-Fe_3_O_4_-mAb nanobot maybe likely a result of the enhanced nuclear accumulation of DOX, as compared to the free DOX.

### Antitumor efficacy of drug loaded nanobots on 3D spheroidal tumors

**Figure 7:**
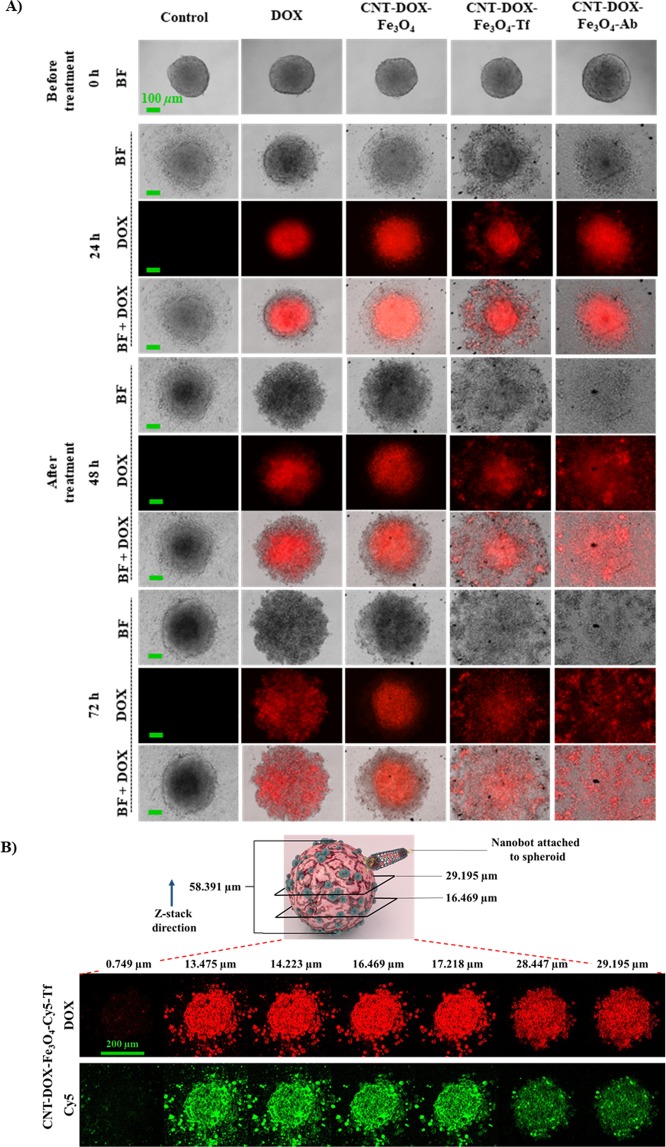
(**A**) Anti-tumor effect of free DOX, CNT-DOX-Fe_3_O_4_ and CNT-DOX-Fe_3_O_4_-Tf on HCT116 spheroids. Red color shows the fluorescence of DOX under an excitation light with a wavelength of 488 nm. After 72 h exposure, CNT-DOX-Fe_3_O_4_-mAb and CNT-DOX-Fe_3_O_4_-Tf were efficacious in tumor-spheroid disintegration and were able to induce significant cell death due to enhanced tumor penetration compared to control (untreated tumor). Scale bar for panel represents 100 µm. (**B**) Deep penetration of CNT-DOX-Fe_3_O_4_-Cy5-Tf NPs into the tumor-spheroid core. Confocal microscopy of spheroid reveals co-localization of DOX (red) and Cy5 tagged CNT-DOX-Fe_3_O_4_-Cy5-Tf NPs (green) at various depths in the tumor mass suggesting deep penetration of the NPs. The schematic depicts the spheroid thickness (58 µm) and the representative planes shown in the confocal image panel below. Scale bar for panel represents 200 µm.

**Figure 8:**
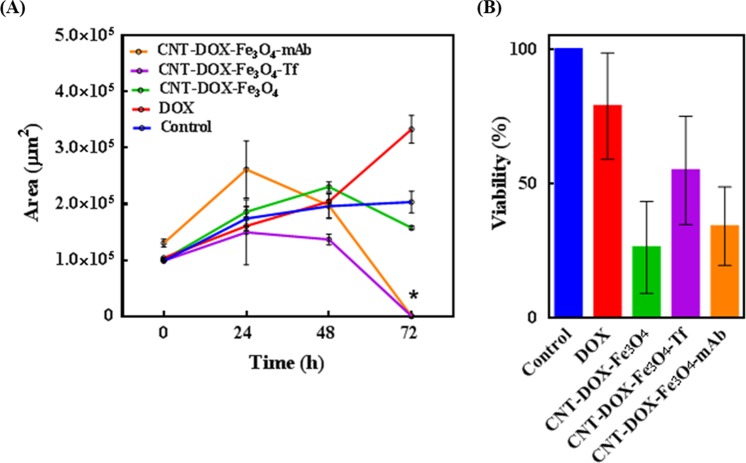
Anti-tumor efficacy of administered nanobots on HCT116 spheroids. (**A**) The tumor mass is expressed as area over a period of 72 h. Tumor disintegration (*) is indicated by reduction in tumor area of spheroids treated with CNT-DOX-Fe_3_O_4_-mAb and CNT-DOX-Fe_3_O_4_-Tf. (**B**) Tumor viability under various treatments are depicted as percent survival, compared to control.

To verify the proposed enhanced tumor penetration of DOX loaded nanobots, multicellular cancer cell 3D spheroids were used to simulate *in vivo* tumors (Fig. 7)^49^. HCT116 spheroids were cultured for 3 days by hanging drop method which promoted 3D tumor formation. The spheroids cultured from single cell suspensions are known to mimic *in vivo* cell-cell interactions *via* formation of inter-cellular junctions contributing to their *in vitro* integrity. Spheroid tumors sustain a balance between cell proliferation and cell death depending on the nutrient supply, DNA replication machinery and death-inducing stimuli. Furthermore, the TME gradient produced due to cellular heterogeneity (outer proliferating layer, followed by a quiescent region and inner necrotic core) is also believed to mimic native tumor physiology, the primary difference being intra-tumor mass vascularization under *in vivo* physiological conditions. *In vivo* tumors are characterized by angiogenesis as a result of complex biochemical interplays to enable tumor survival *via* vascularization^50,51^. Lab-grown spheroids thus, have to rely on surrounding media for nutrient supply. Consequently, as a result of nutrient gradient, the spheroids develop cellular heterogeneity as described above. As the cells proliferate, the number of dead cells accumulates as well, especially in the necrotic core of the spheroid leading to the formation of a dense inner core and an outermost scattered mono layer of shed cells (Fig. 7A, control). Since free DOX can be rapidly taken up by the outer layer of the spheroid cells, a pronounced DOX effect was observed initially. However, DOX effect dissipated in the cell medium as a relatively dilute anti-neoplastic drug, DOX was apparently not sufficient to induce death of remaining cells even after 72 h (Fig. 7A). Note that the inner dense (darker) core is reduced, despite the apparent growth of the tumor area. The widening of the tumor base is attributed to the reduced cell-cell adhesions resulting from DOX treatment which consequently undermines the integrity of the spheroid mass causing it to settle downward and spread. CNT-DOX-Fe_3_O_4_-mAb and CNT-DOX-Fe_3_O_4_-Tf nanobots were significantly more efficacious in tumor reduction than free DOX and the CNT-DOX-Fe_3_O_4_ nanobot. CNT-DOX-Fe_3_O_4_-mAb and CNT-DOX-Fe_3_O_4_-Tf nanobots were able to induce cell death resulting in tumor spheroid disintegration compared to control after 72 h of treatment, as shown in Fig. 7A. The lack in spheroid cohesion is apparent from 48 h for both treatments, while the inner dense cores were abolished completely by 72 h. On the other hand, the control tumor after 72 h depicted an increase in core area by ~104% (from ~9.8 × 10^4^ to ~20.3 × 10^4^ µm^2^) and CNT-DOX-Fe_3_O_4_ nanobot treated tumor by ~62% (from ~9.9 × 10^4^ to ~15.8 × 10^4^ µm^2^) compared to their respective before treatment area. One reason for the enhanced efficacy may be the deep tumor penetration ability of CNT-DOX-Fe_3_O_4_-Tf and CNT-DOX-Fe_3_O_4_-mAb due to the forward thrust obtained by nanobots and the delayed DOX clearance at the tumor site because of the retention property of the NPs. DOX is also responsible for inhibiting/blocking the transcriptome^43^, which may also affect the cancer cells ability to maintain the cell adhesion/cell contact machinery. It is conceivable that sustained DOX exposure may reduce the ability of spheroid cells to self-adhere/assemble and be subject to disaggregation and thus be increasingly more prone to the cytotoxic effects of DOX. The effects of CNT-DOX-Fe_3_O_4_-Tf and CNT-DOX-Fe_3_O_4_-mAb appear to manifest in a manner consistent with the above statement (Fig. 7A). Additionally, deep tumor penetration of CNT-DOX-Fe_3_O_4_-Cy5-Tf nanobots into the tumor-spheroid core was studied using confocal microscopy. Z-stack images of the spheroids revealed co-localization of DOX (red) and Fe_3_O_4_-Cy5 (green) signals at various planes, suggesting deep penetration of NPs as well as their internalization into individual tumor cells (Fig. 7B). The DOX and Fe_3_O_4_-Cy5 signals were visible with substantial intensity up to the core (~29 µm) of the entire tumor mass (~58 µm). The ablation of the dense tumor cores (Fig. 8A) are indicative of exposure to CNT-DOX-Fe_3_O_4_-Tf and CNT-DOX-Fe_3_O_4_-mAb particles. Furthermore, the protection of CNT-encapsulated DOX against rapid drug efflux prior to endocytosis and the subsequent intracellular release of DOX may contribute to the enhanced antitumor effect. In contrast, free DOX and CNT-DOX-Fe_3_O_4_ were less effective in tumor regression possibly as a result of the small size of free DOX or extracellularly released DOX that would be rapidly diffused away from the tumor interstitium. Interestingly, tumor viability studies demonstrated a greater anticancer activity for CNT-DOX-Fe_3_O_4_ particles (Fig. 8B), compared to the targeted NPs. This may result from sustained non-specific TME-acid triggered Fe_3_O_4_ uncapping and DOX release in the immediate vicinity and interior of the spheroid. The resultant system is hypothesized to have generated a very high localized DOX concentration in the TME resulting in significant cell death, but insufficient impact to destroy the tumor integrity. Tf and anti-EpCAM mAb conjugated nanobots exhibited greater cell surface targeting, however this delayed the release of DOX payload intracellularly. The effect can be attributed to surface epitope interactions between Tf as well as anti-EpCAM mAb nanobot ligands and over-expressed cell surface receptors.

Finally, although Tf and anti-EpCAM mAb conjugated nanobots achieve greater targeting their cellular internalization mechanism, release from the overexpressed cell surface receptors, and the release of DOX inside the cells seems to be delayed. This can be attributed to the specific and tight interactions between over-expressed cell surface receptors and the Tf as well as anti-EpCAM mAb nanobots. However, the self-propulsion, cell surface specificity, cell kinetics, and finally the anticancer activity measurements makes these nanobots interesting to be further explored in anticancer therapy.

## Conclusions

We have demonstrated a novel self-powered multifunctional gated nanobot that offers promising alternative drug delivery system based on rapid autonomous motion for quicker and deeper delivery to the tumor site. The nanobots were fabricated by chemically coordinating and conjugating multiple components such as Fe_3_O_4_ NPs and targeting moiety such as Tf or anti-EpCAM mAb to CNT. This nanobot system combines several intriguing features, namely self-propulsion, high DOX loading, tumor targeting and profound penetration ability, *in situ* pH triggered release of the DOX, and improved drug availability. The CNT-DOX-Fe_3_O_4_-Tf nanobots demonstrated ultrafast self-propulsion (0.972 and 0.535 mm s^−1^) not only in high ionic media (PBS buffer) but also in biological media such as DMEM (2.333 and 1.120 mm s^−1^) and blood serum (8.026 and 1.120 mm s^−1^), a crucial ability necessary for its use in biomedical applications. The speed of the nanobot in serum was ~8.3 and ~3.4 times the speed seen in PBS and DMEM. The driving force of 592, 1304 and 5435 pN for the nanobot’s upward propulsion was significantly higher. The high driving force and thus higher speed of CNT-DOX-Fe_3_O_4_-Tf nanobot in serum is maybe due to adsorbed serum catalase enzyme which may be imparting additional propulsion by catalytic property and thus enhancing generating of oxygen bubbles. Thus, propulsion of nanobot was also observed in serum with no external H_2_O_2_ indicating ability of the nanobot to propel in blood and penetrate tumor by utilizing H_2_O_2_ present in the TME. The cellular uptake study showed controlled release of DOX due to opening of pH-sensitive nanogates by cleavage of amide bond in the acidic lysosomal compartments. Further, higher intensity of DOX in nucleus for CNT-DOX-Fe_3_O_4_-Tf nanobot indicated not only efficient and steady release of DOX but also superior retentive property of the nanobot carriers. Upon administration to tumor spheroids, CNT-DOX-Fe_3_O_4_-Tf and CNT-DOX-Fe_3_O_4_-mAb nanobots were significantly more efficacious in tumor reduction at 72 h than the control groups including free DOX and CNT-DOX-Fe_3_O_4_ nanobot. One reason for the enhanced efficacy might be the profound tumor penetration ability due to the propulsion of CNT-DOX-Fe_3_O_4_-Tf and CNT-DOX-Fe_3_O_4_-mAb nanobots, and the delayed clearance at the tumor site because of the retention property of the NPs. Thus, the synthesized CNT-DOX-Fe_3_O_4_-mAb and CNT-DOX-Fe_3_O_4_-Tf nanobots would be effective in smaller numbers, designed to selectively and efficaciously deliver the drug payload in targeted cancer cells alone within the TME.

## Materials and methods

### Reagents

Multi-walled carbon nanotubes (CNTs) having outer diameter of 10–15 nm; length 1–5 µm; and purity >99%, were purchased from Ad-Nano Technologies, India. Ferric chloride tetrahydrate, ferrous chloride hexahydrate, transferrin (Tf), *N*-(3-Dimethylaminopropyl)-*N*′-ethylcarbodiimide (EDC.HCl), glutathione (GSH) and horeseradish peroxidase (type VI) were purchased from Sigma-Aldrich, USA. Doxorubicin hydrochloride (DOX) was received as a gift from Naprod Life Sciences, India. Cy5 mono NHS ester was procured from GE Healthcare UK Limited, and LysoTracker Green DND-26 was procured from Invitrogen, Thermo Fisher Scientific. HCT116 cells were obtained from the National Centre for Cell Science, India. McCoy’s 5A, fetal bovine serum (FBS), Penicillin and streptomycin were purchased from Sigma-Aldrich, USA. Ultrapure water (MilliQ) acquired from a Merck Millipore system, Germany, was used throughout. All other chemicals procured were of analytical grade and utilized without further purification.

### Functionalization of CNTs (CNT-COOH)

CNT was purified and oxidized using a modified literature procedure^52^. In brief, 85 mg of CNT was dispersed in 100 mL mixture of H_2_SO_4_/HNO_3_ (3:1) and then sonicated for 6 h. The mixture was diluted with 100 mL ice cold water, concentrated by centrifugation and washed with 5% NaOH solution and ultra-pure water. Resulting functionalized CNT was dried at 80 °C (12 h).

### Synthesis of Fe_3_O_4_-GSH

Fe_3_O_4_ NPs were prepared by co-precipitation of ferric and ferrous ions (2:1) using aqueous ammonium hydroxide solution and then heated at 80 °C for 30 min, washed for several times with ultra-pure water and ethanol and finally dried at 70 °C (4–6 h)^53^. 5 mg of Fe_3_O_4_ NPs were dispersed in 150 µl of ultra-pure water and 50 µl of methanol and sonicated for 15 min. 4 mg of GSH was dissolved in 50 µl of ultra-pure water, added in above solution and again sonicated for 2 h. The GSH functionalized NPs were then isolated by magnetic separation, washed repeatedly with ultra-pure water and dried well^54^.

### Loading of DOX in CNT-COOH (CNT-DOX)

Loading of DOX in CNT-COOH was carried using a modified procedure previously reported by us^24^. Briefly, 20 mg of CNT-COOH were suspended in 5 mL solution of DOX (8 mg/mL). The solution was sonicated for 6 h and was allowed to stand for further 12 h. The synthesized product, CNT-DOX was collected by centrifugation and dried well at room temperature.

### Synthesis of CNT-DOX-Fe_3_O_4_

20 mg of CNT-DOX and 5 mg of EDC were added in 5 ml of phosphate buffer (pH 7.4) and then agitated for 30 min. 20 mg of Fe_3_O_4_-GSH was added in the same mixture and agitated for another 1 h. The conjugated CNT-DOX-Fe_3_O_4_ NPs were magnetically separated, washed extensively with phosphate buffer to remove externally adsorbed DOX and then dried well at 40 °C.

### Synthesis of CNT-DOX-Fe_3_O_4_-Tf

10 mg of CNT-DOX-Fe_3_O_4_ were treated with 1 mL of EDC and NHS solution (50 mm each solution in phosphate buffer (pH 7.4). After 30 min of agitation, CNT-DOX-Fe_3_O_4_ NPs were separated with magnet and washed with PBS (3 times). 1 mL of Tf solution (5 mg/mL) was added. The reaction was then agitated for 4 h. The synthesized product, CNT-DOX-Fe_3_O_4_-Tf NPs were collected by magnetic separation and dried well at room temperature. Similarly, conjugation of anti-EpCAM mAb to CNT-DOX-Fe_3_O_4_ NPs was carried out.

### Characterization

TEM analysis was carried out using Tecnai FEI G2 (accelerating voltage of 300 kV). The samples were prepared by placing a drop of CNT-DOX-Fe_3_O_4_-Tf suspensions (in DI water) onto a Formvar-covered copper grid. The water was allowed to evaporate in air at room temperature before imaging. FTIR spectral studies were carried out using a Perkin Elmer Fourier Transform Infrared (FTIR) spectrometer, USA in the range between 4000 and 400 cm^−1^, with a resolution of 2 cm^−1^. The UV-Vis absorption spectra were recorded on Agilent Technologies Cary 60 UV spectrophotometer.

### Catalytic activity of Fe_3_O_4_ in H_2_O_2_

The catalytic activity of Fe_3_O_4_ in H_2_O_2_ was evaluated by incubating a 500 µg/mL dispersion of Fe_3_O_4_ in PBS pH 7.4 with various concentrations of H_2_O_2_ (0.006 w/v% to 0.05 w/v%) for 30 min. The difference in initial concentration of H_2_O_2_ and the concentration of H_2_O_2_ after 30 min was used to determine the rate of reaction. The concentration of H_2_O_2_ in solution was determined using a modified horseradish peroxidase (HRP) based colorimetric assay^55^. Briefly, 10 µL of test sample (either standard H_2_O_2_ solutions for calibration curve or reaction samples) was added to 990 µL of an enzyme mixture and incubated for 30 min in dark. The enzyme mixture comprised of 500 µL of 84 mM phosphate buffer pH 7, 350 µL of 12 mM phenol, 100 µL of 0.5 mM 4-aminoantipyrene and 40 µL of 1 U/mL of HRP in 84 mM phosphate buffer pH 7. The absorbance was read at 505 nm.

### Motion behavior of nanobot in different fluids

The self-propulsion of the CNT-DOX-Fe_3_O_4_-Tf nanobot in PBS, DMEM and serum with different concentrations of H_2_O_2_ (0, 0.05, 0.1, 0.5, 1, 2, 4, 6 and 8%), was recorded with Dino-Lite digital microscope at 50× magnification, using the Dino-Capture 2.0 v (https://www.dino-lite.com/). This was then processed to convert in to Avi format using Format Factory and chosen best clip using VirtualDub 1.10.4 v (http://www.virtualdub.org/). The propelling microparticles were tracked and calculated its speed using MTrackJ plugin from ImageJ 1.8.0_112v (https://imagej.net/MTrackJ).

### Drug release profiles of the nanobot

pH dependent *in vitro* release profile of DOX from CNT-DOX- Fe_3_O_4_-Tf was evaluated by suspending 10 mg of material in 20 ml of pH 5 and pH 7.4 phosphate buffer. The nano system was stirred continuously at ambient temperature. 1 ml of aliquot was withdrawn at different time intervals, centrifuged and was analyzed using UV spectroscopy at λ_max_ of 484 nm. 1 ml of fresh phosphate buffer of same pH was replaced at every time point in the dissolution media. All the experiments were performed in triplicate.

### Cell culture

HCT116 was procured from NCCS and cultured in McCoy’s 5A, supplemented with 10% fetal bovine serum and 100 unit/ml penicillin, 100 mg/ml streptomycin and maintained in CO_2_ incubator at 37 °C and 5% CO_2_ saturation.

### Nanobot’s efficacy as drug delivery vehicle

The cytotoxic activity of compounds was quantitatively determined by a colorimetric assay utilizing (3-(4, 5-dimethylthiazol-2-yl)-2, 5- diphenyltetrazolium bromide) (MTT). HCT116 cells were seeded in 96-well plates (5000 cells/well) and maintained in CO_2_ incubator for 24 h at 37 °C in McCoy’s 5A medium supplemented with 10% FBS and 1% antibiotics. The free DOX, CNT-COOH, CNT-Fe_3_O_4_, CNT-DOX-Fe_3_O_4_-Tf and CNT-DOX-Fe_3_O_4_-mAb nanobots were added in the wells and incubated for 48 h. The DOX concentration in the study was 0.377 µg/ml (IC50). The cells were then incubated with MTT for 4 h at 37 °C. In the viable cells mitochondrial succinic dehydrogenase reduced MTT to an insoluble formazan precipitate. After removal of the media, dimethylsulfoxide (DMSO) was added to each well. After complete solubilization of the purple MTT formazan (approximately 10–15 min), the absorbance was measured at 570 nm with a microplate reader on Infinites F200 PRO (Tecan, Austria). Background readings (blank) were obtained from cell-free wells containing media also incubated with the MTT solution.

### Time dependent cellular entry studies using fluorescence microscopy

5000 cells of HCT116, were seeded in each well of 96 well plate. After 24 h, cells were treated with free DOX, CNT-DOX-Fe_3_O_4_ and CNT-DOX-Fe_3_O_4_-Tf nanobots in a time dependent manner (1 h, 4 h, 24 h and 48 h). The concentration of DOX was 0.377 µg/ml (IC50). The free DOX and all the nanobots were added according to the IC50 value of DOX and the DOX loading (60 µg/mg) in the nanobots. The media were removed and cells were washed with phosphate buffered saline (PBS) after consecutive time points and processed for fluorescence microscopy. Cells were fixed with 4.0% (w/v) paraformaldehyde for 15 min at room temperature, then washed with PBS and maintained in PBS. Cells were stained with 4,6-diamidino-2-phenylindole (DAPI) (Sigma) and examined under a fluorescence microscope (Carl Zeiss, AxioObserver A3, USA).

Additionally, the co-localization of DOX in acidic lysosomal compartments with LysoTracker green as a fluorescent probe was studied using confocal laser scanning microscopy (CLSM), Leica Microsystems.

#### Time dependent cellular entry studies using zeta potential

HCT116 cells were incubated with CNT-DOX-Fe_3_O_4_-Tf nanobots at pH 7.4 and 6.5 in presence or absence of H_2_O_2_ (4.98 mm). The 5000 cell were re-suspended in1 mL of 40 mm HEPES buffer pH 7.4 and 6.5. The zeta potential values of HCT116 cells and cells incubated with CNT-DOX-Fe_3_O_4_-Tf for different time duration *viz*. 0 min and 24 h, were measured using Zetasizer Nano ZS (Malvern Instruments, Worcestershire, UK). All the Zeta (ξ) potential measurements were carried out at room temperature using phase analysis light scattering mode.

### Culture of HCT116 cell 3D spheroidal tumor

3D tumor spheroids were formed by a modified method of the hanging drop technique^49^. In brief, the lid of sterile 12 well plates were coated with poly(dimethoxysiloxane) (PDMS) and Sylgard 184 in a 10:1 ratio and cured at 80 °C for around 45 min. The lids were then placed under UV for 30 min to ensure sterility of the PDMS coated surface. HCT116 cell suspension was prepared in complete McCoy’s 5A medium. 20 μL drops of the cell suspension with a density of 2,500 cells/drop were placed at regular intervals on the PDMS coated lid. The wells were filled with sterile MilliQ water to ensure hydration of drops upon incubation. Thereafter, the cells were incubated at 37 °C in presence of 5% CO_2_ for three days. Finally, the coherent mass of 3D tumor spheroids formed was selected for further studies.

### Antitumor efficacy of drug loaded nanobots

Tumors generated by hanging drop method were transferred to 96 well plate for treatment with DOX and nanobots. The 3D tumor spheroids upon transfer to 96 well plate were immediately treated with free DOX (5 µg/mL) and nanobots containing equivalent DOX for 72 h. The images of tumors were captured using Carl Zeiss, AxioObserver A3, USA, USA inverted fluorescence microscope. The exposure time while capturing bright field images was fixed at 100 ms and the exposure time while capturing fluorescence images was fixed at 400 ms.

Furthermore, the viability of tumors after 72 h was analyzed by MTT assay following similar protocol mentioned earlier. Similarly, for CLSM the 3D tumor spheroids were transferred to a glass bottom well plate before capturing z-stack images. The z-stack images were captured at intervals of 0.75 µm.

## Electronic supplementary material

Supplementary Material.
