# Supplementary material for: Self-Propelling Targeted Magneto-Nanobots for Deep Tumor Penetration and pH-Responsive Intracellular Drug Delivery
Source: Sci Rep. 2020 Mar 13;10:4703. doi: 10.1038/s41598-020-61586-y (PMC7070039; doi:10.1038/s41598-020-61586-y)
Supplement: Supplementary file 1 — Supplementary Material. [file 41598_2020_61586_MOESM1_ESM.pdf]

Self-Propelling Targeted Magneto-Nanobots for Deep Tumor Penetration and pH-Responsive Intracellular Drug Delivery

Saloni S. Andhari,[1,4] Ravindra D. Wavhale,[2,4] Kshama D. Dhobale,[2] Bhausaheb V. Tawade,[2] Govind P. Chate,[2] Yuvraj N. Patil,[2] Jayant J. Khandare[3]* and Shashwat S. Banerjee[2]*

[1] Maharashtra Academy of Engineering Education and Research's Maharashtra Institute of Pharmacy, Pune 411038, India.

[2] Maharashtra Institute of Medical Education and Research, Talegaon Dabhade, Pune 410507, India.

[3] School of Pharmacy, Dr. Vishwanath Karad MIT World Peace University, Pune 411038, India.

[4]These authors contributed equally: Saloni S. Andhari and Ravindra D. Wavhale

*email: jayant.khandare@mippune.edu.in; shashwatbanerjee@mitmimer.com

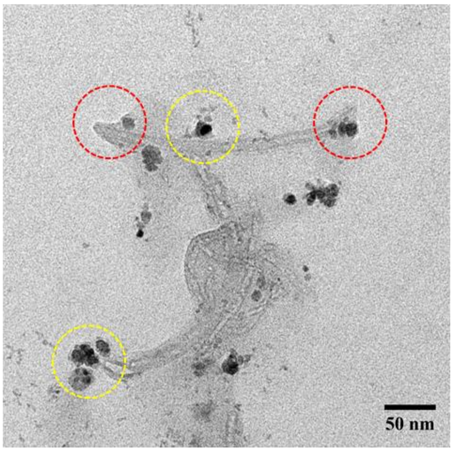

Figure S1. TEM microscopy images of CNT-DOX-Fe_3O_4-Tf nanobots with Fe_3O_4 NP caps at both the ends of CNT (indicated with dashed circles for each CNT).

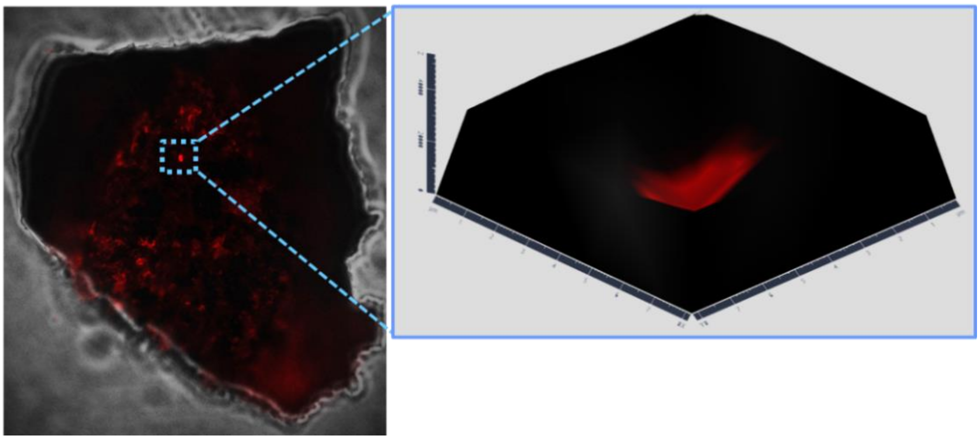

Figure S2. 2.5D microscopy imaging of the CNT-DOX-Fe_3O_4-Tf particles, highlighting the presence of DOX (red) within the nanopores of the CNT carrier particle (gray).

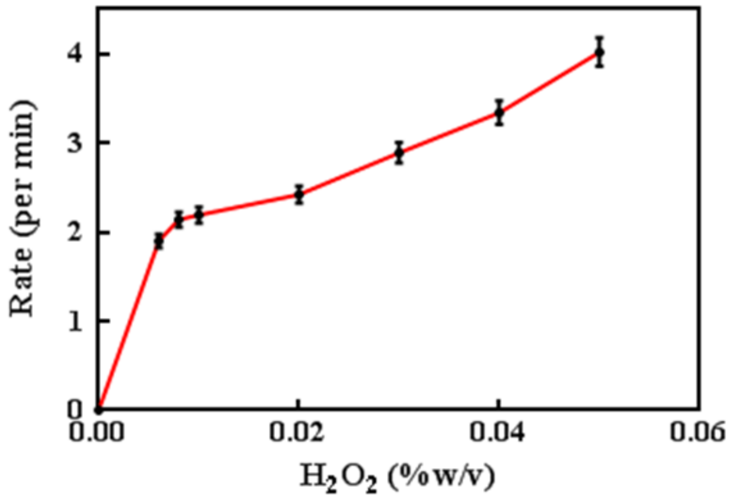

Figure S3. Catalytic ability of Fe_3O_4 towards H_2O_2 at various concentrations of H_2O_2 in PBS pH 7.4.

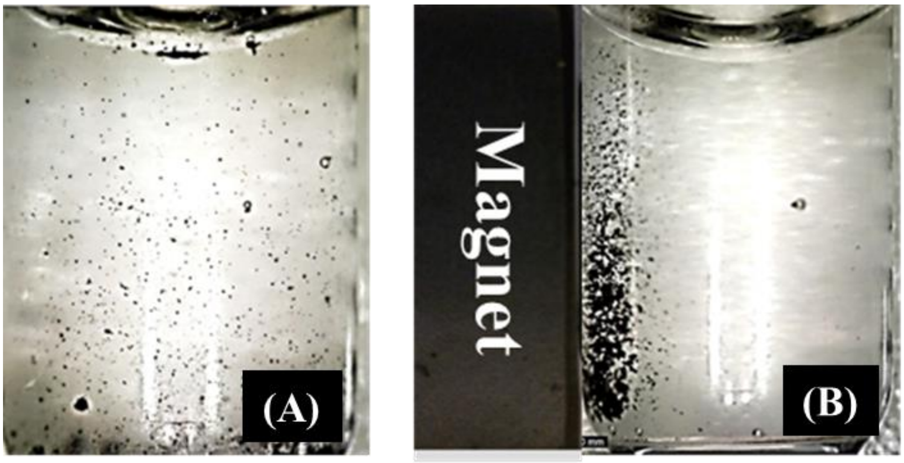

Figure S4. (A) CNT-DOX-Fe_3O_4-Tf nanobot with excellent dispersibility and propulsion in PBS containing H_2O_2. (B) CNT-DOX-Fe_3O_4-Tf nanobot also can be drawn from the solution to the sidewall of the vial under magnetic guidance. The images were captured using Dino-Lite digital microscope at 50X magnification, using the Dino-Capture 2.0v (https://www.dino-lite.com/).

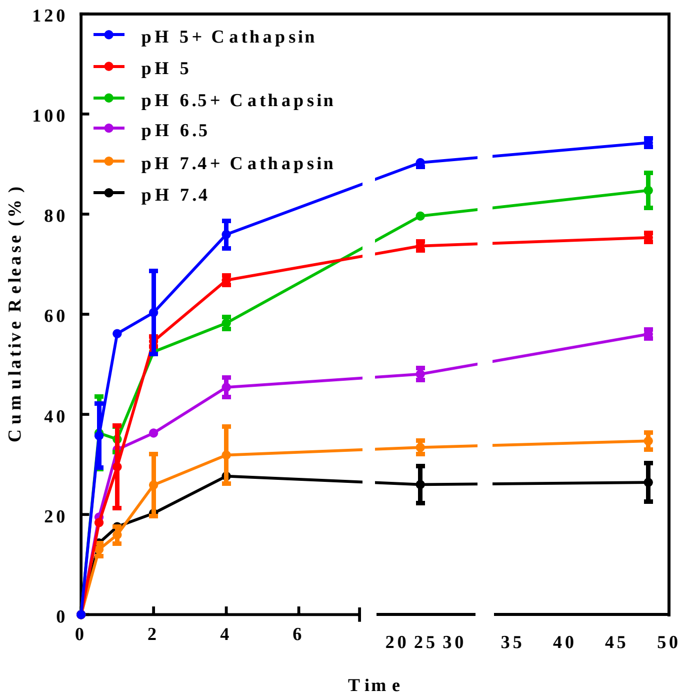

Figure S5. Time dependent cumulative release of DOX from CNT-DOX-Fe_3O_4-Tf. Significantly high release of DOX from CNT-DOX-Fe_3O_4-Tf in presence of cathapsin B at pH 5 compared to pH 6.5 and 7.4 was observed due to degradation of amide linkage resulting in time-dependent uncapping of CNT. Low release of DOX at pH 7.4 highlighted efficient trapping of DOX in the CNT cavity by with Fe_3O_4 NPs cap.

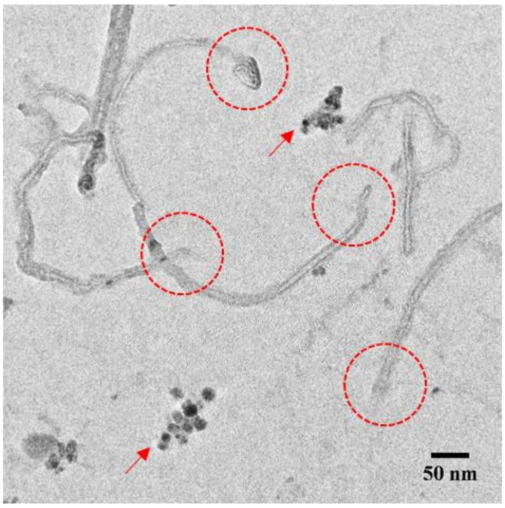

Figure S6. TEM microscopy image of CNT-DOX-Fe_3O_4 nanobots after release study in phosphate buffer of pH 5.0 containing cathepsin B. Circles in red indicate the terminal ends of CNTs devoid of Fe_3O_4 NPs, while the red arrows indicate free Fe_3O_4 NPs separated from the CNTs.

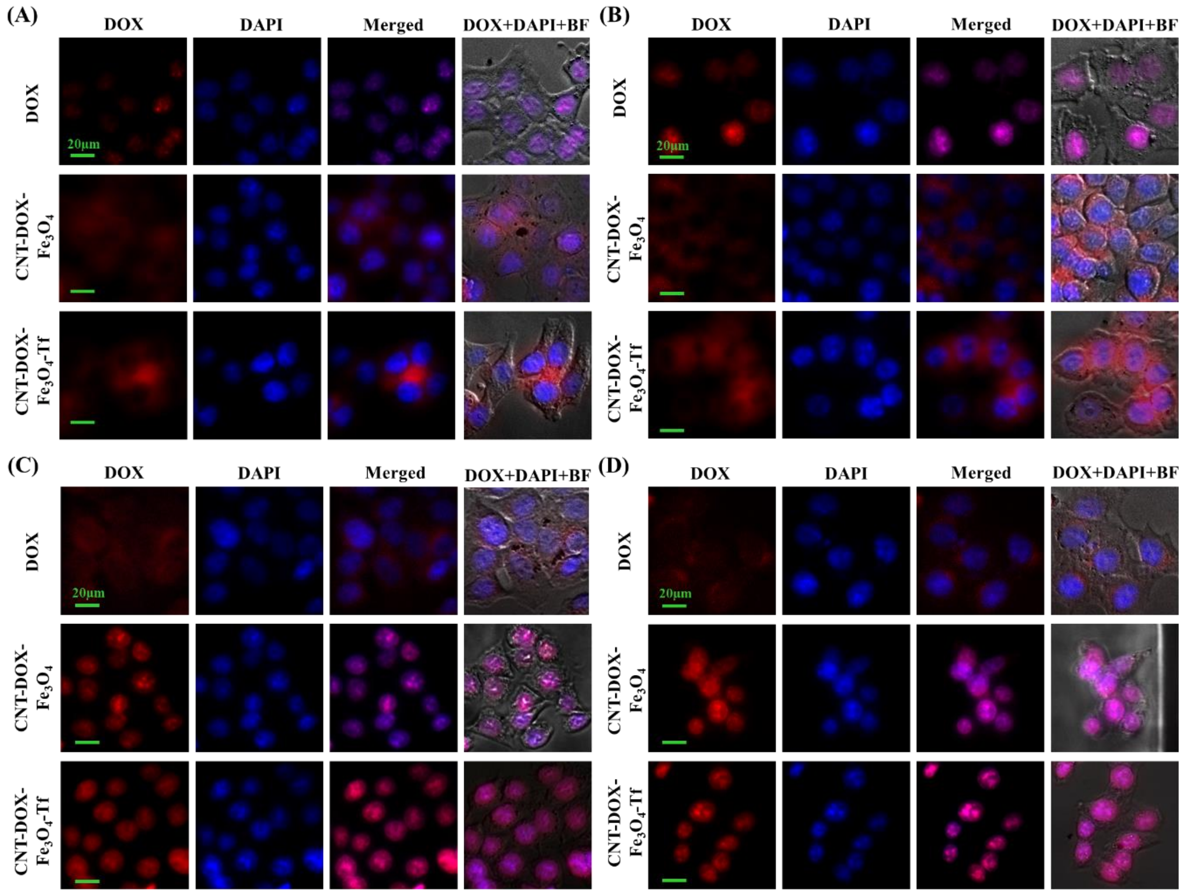

Figure S7. Fluorescent images of HCT116 cells treated with free DOX, CNT-DOX-Fe_3O_4 and CNT-DOX-Fe_3O_4-Tf nanobots. (A) At 1 h exposure and in pH 7.4, free DOX in the cell was higher than that of CNT-DOX-Fe_3O_4-Tf. On the other hand, the intensity of DOX released from CNT-DOX-Fe_3O_4 was higher than CNT-DOX-Fe_3O_4-Tf. (B) At 1 h exposure and in pH 6.5, the CNT-DOX-Fe_3O_4-Tf nanobot demonstrated increase in cellular drug content in the acidic pH of 6.5. (C) At 48 h and in pH 7.4, most of the DOX is released from CNT-DOX-Fe_3O_4-Tf suggesting the efficient expulsion of DOX from interior cavity of CNT after opening of Fe_3O_4 nanolid in lysosomal conditions. DOX released from CNT-DOX-Fe_3O_4-Tf nanobot co-localized with DAPI concentrated in the nuclear region highlighting the nucleosome bodies, which contain the chromatin matter. (D) At 48 h and in pH 6.5, the co-localization of nuclear region effect was more pronounced suggesting preferential binding of

DOX to DNA and nucleosome-bound topoisomerases. Cell shrinkage/disaggregation was observed due to DOX cytotoxicity > 48 h. (Scale bar = 100 μm).

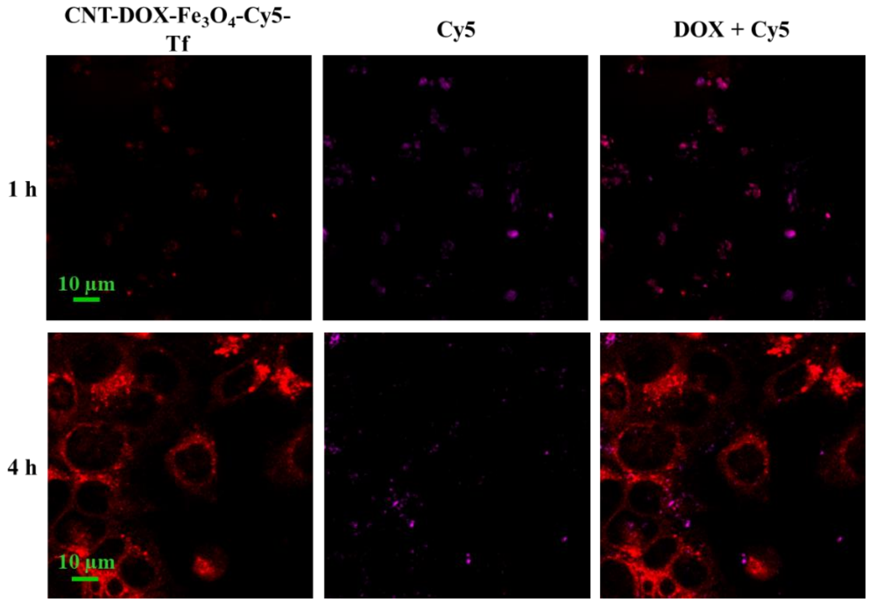

Figure S8. Disengagement of Fe_3O_4 NPs resulting in uncapping of CNT-DOX-Fe_3O_4-Cy5-Tf nanobots and cellular release of DOX. Strong localization of Cy5 (purple, Fe_3O_4 NPs) with DOX (red) at 1 h indicate site-restriction of DOX within CNT-DOX-Fe_3O_4-Cy5-Tf NPs. 4 h post-treatment images reveal separation of Fe_3O_4 and DOX signals, consistent with detachment of Fe_3O_4 caps from CNT and subsequent release of DOX from CNT. Scale bars indicate 10 µm.

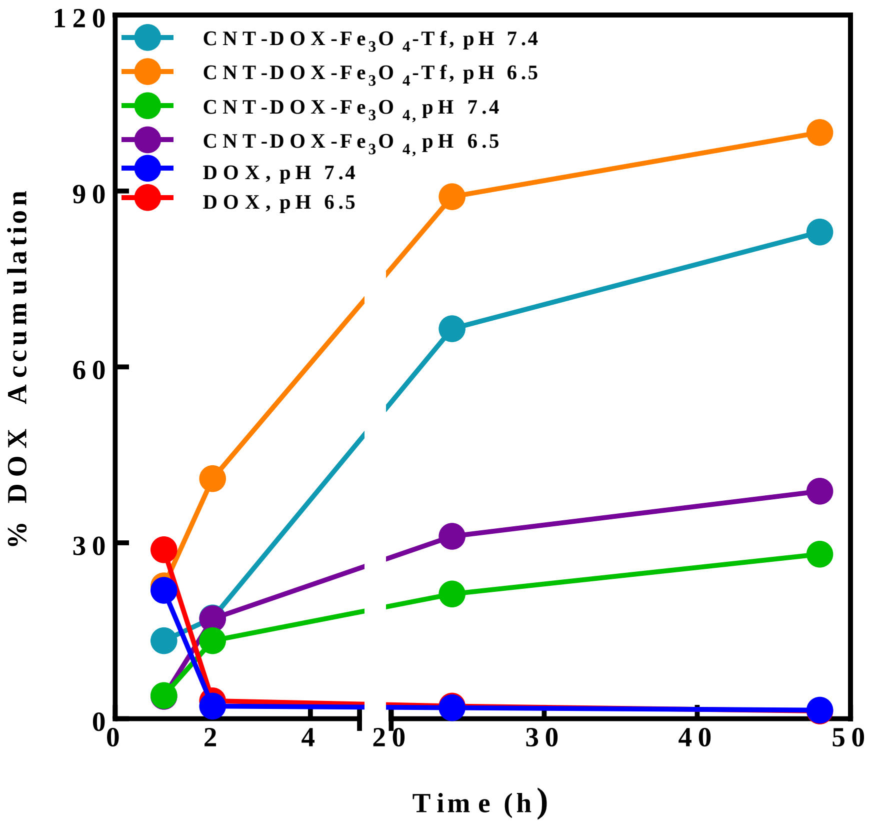

Figure S9. Intracellular DOX accumulation upon treatment with nanobots at varying pH.
